# Hospital Meetings

**Published:** 1900-12-08

**Authors:** 


### HOSPITAL MEETINGS.

### SURGICAL AID SOCIETY.

The thirty-eighth annual meeting of the Surgical Aid Society was held at Cannon Street Hotel on Monday, 3rd inst., the Lord Mayor (Mr. Alderman Green) presiding. The secretary, Mr. R. C. Tresidder, read the report, which stated that the position held by the Society in public estimation was indicated by the fact that the finances had been well sustained, despite the various national funds which during the year had claimed so much charitable support and had involved serious declension in the incomes of many institutions. The net income for the past year was £17,009, as against £17,157 in 1899, a decrease of £148; but, excluding the item of legacies, which had diminished from £3.530 to £907, the total income showed an increase of £2,474 upon that of the preceding year. This the committee regarded as eminently satisfactory, especially in view of the fact that the annual subscriptions had increased from £8,702 to £8,942—an advance of £240. Of the total ordinary expenditure of £14,695, exclusive of investments and office extension, no less than £12,191 was on the items of surgical appliances and surgeons' fees, so that 82·95 per cent. of the expenditure was for actual relief, and only 17·05 per cent. for management and office expenses. The committee were also thankful to report the receipt during the period of a munificent bequest of £20,614 from the late Mr. Edward Mackeson, of Hyde Park Square. They had under their serious consideration several proposals for dealing with this bequest in the best interests of the Society and of the suffering poor.

The number of patients relieved during the year was 16,714—a weekly average of 321. 25,219 appliances were thus supplied, while 320 articles, such as water and air beds and invalid carriages, were lent in cases of temporary illness or disablement. Great benefit had been felt by all participating in the practical part of the work of the Society from the recent enlargement and rearrangement of the offices and the enhanced facilities afforded thereby. Mr. T. H. Openshaw, the junior surgeon, who was selected as surgeon-in-charge of the Imperial Yeomanry Field Hospital, had now returned and resumed his duties. With regret the committee recorded the loss of two of their members during the year. Mr. J. Albert Causton resigned in November 1899, after holding office for sixteen years; Mr. James Corry Sherrard, who had been a member fourteen years, died on March 4th last. The places of these gentlemen had been filled by Mr. H. J. Powell and Dr. H. G. Thompson—the latter in recognition of his services as secretary of the Croydon Branch since its establishment in 1884.

The chairman moved the adoption of the report in a resolution pledging the meeting to continued support of the work of the Society. He congratulated the committee on their policy and on the progress they were making. There were very few institutions doing a more useful work. When the Society was established, in 1862, there was nothing else of the sort in existence; and since that time—less than forty years ago—339,219 appliances had been distributed. By making special grants, the committee provided that no needy applicant went unrelieved. The Society was in every way worthy of their best endeavours and their hearty commendation.

The resolution was seconded by the Rev. R. S. de C. Laffan, who pointed out that the first year's income of the Society from annual subscriptions was only £102, while now it totalled close on £9,000 per annum. The report was adopted. On the motion of Mr. Herbert Tritton, seconded by Dr. H. G. Thompson, a vote of thanks was accorded to Mr. William Grey, the treasurer, who was prevented by illness from being present. The committee was re-elected; and votes of thanks to the surgeons and local officials, and to the Lord Mayor for presiding, terminated the proceedings.

